# Common endosymbionts influence host sexual selection by shaping mating preferences via altered chemical communication

**DOI:** 10.1093/evlett/qraf044

**Published:** 2025-12-08

**Authors:** Amir H Tourani, Alihan Katlav, James M Cook, John Hunt, Shawan Karan, Markus Riegler

**Affiliations:** Hawkesbury Institute for the Environment, Western Sydney University, Locked Bag 1797, Penrith, NSW 2751, Australia; Hawkesbury Institute for the Environment, Western Sydney University, Locked Bag 1797, Penrith, NSW 2751, Australia; Hawkesbury Institute for the Environment, Western Sydney University, Locked Bag 1797, Penrith, NSW 2751, Australia; School of Science, Western Sydney University, Locked Bag 1797, Penrith, NSW 2751, Australia; Hawkesbury Institute for the Environment, Western Sydney University, Locked Bag 1797, Penrith, NSW 2751, Australia; Hawkesbury Institute for the Environment, Western Sydney University, Locked Bag 1797, Penrith, NSW 2751, Australia

**Keywords:** endosymbiont, symbiont-host conflict, mate choice, assortative mating, chemical communication, cuticular hydrocarbons

## Abstract

Maternally transmitted endosymbionts of arthropods are common and phylogenetically diverse. Several bacteria, including *Wolbachia* and *Cardinium*, have independently evolved the ability to induce cytoplasmic incompatibility (CI) limiting the reproduction in females lacking the endosymbionts carried by their mates. While promoting endosymbiont spread, CI is costly to endosymbiont-free females. Such host-endosymbiont conflicts are expected to affect host mating preferences, yet this has scarcely been studied in hosts carrying multiple, potentially competing, endosymbionts. We investigated mate choice and chemical communication in a significant pest of citrus, Kelly’s citrus thrips (*Pezothrips kellyanus*), naturally carrying CI-inducing *Cardinium* and *Wolbachia*. Unlike females with both endosymbionts (CW) that had no preference for males with particular endosymbiont associations, females with only *Cardinium* (C) preferred compatible C and endosymbiont-free males over incompatible CW males. In contrast, endosymbiont-free females showed no preference, despite experiencing similar CI risks when facing incompatible C and CW males. Male mating success, however, mostly depended on female receptivity and not on endosymbiont association. Furthermore, chemical analyses revealed that males with different endosymbiont associations had distinctly different cuticular hydrocarbon (CHC) profiles, with the CHC profile of CW males markedly including tridecane, a compound known to influence animal behavior. The results indicate that *Cardinium* enables females to avoid *Wolbachia*-induced CI based on the distinct chemical cues of incompatible males. Our findings highlight the role of common endosymbionts and their interactions in sexual selection through their effects on chemical and behavioral traits of hosts, emphasizing the importance of these factors in endosymbiont and host population dynamics, as well as endosymbiont-based pest control strategies.

## Teaser

Sexual selection drives the evolution of traits that enhance reproductive success. While such traits are shaped by genetic and environmental factors, microbial symbionts can also play a role. Our study demonstrates that common maternally transmitted endosymbionts of arthropods mediate sexual selection by affecting mating preferences and chemical communication of their host. *Cardinium* enabled females to preferentially mate with compatible males thereby avoiding the risk of infertility arising from matings with incompatible males carrying *Wolbachia*. The males’ endosymbiont association status was linked to distinct cuticular hydrocarbon profiles, which may assist females to recognize their compatibility. Such multipartite interactions are important as they can affect the population dynamics of both endosymbionts and hosts, as well as the effectiveness of endosymbiont-based pest control.

## Introduction

Sexual selection drives the evolution of traits that enhance reproductive success via mate choice and competition (Anderson, 1994). Besides the genetic and environmental factors that shape such traits, microbial symbionts can also influence sexual selection by altering host reproduction and mating behaviors (Wedell, 2020). Among these symbionts, *Wolbachia* (Alphaproteobacteria) and *Cardinium* (Cytophagia), two phylogenetically diverged and maternally transmitted endosymbiotic bacteria common in arthropods, are particularly notable for their ability to affect host reproduction, such as by inducing cytoplasmic incompatibility (CI) (Doremus & Hunter, 2020; Kaur et al., 2021; Mathieson et al., 2025; Werren et al., 2008). Other maternally transmitted endosymbionts that also induce CI, *Lariskella, Mesenet, Rickettsia* (Alphaproteobacteria), *Rickettsiella* (Gammaproteobacteria) and *Spiroplasma* (Mollicutes) have been discovered more recently (Owashi et al., 2024; Pollmann et al., 2022; Rosenwald et al., 2020; Takano et al., 2021; Umanzor et al., 2025). CI occurs when endosymbiont-free females mate with males carrying CI-inducing endosymbionts, resulting in embryonic mortality of fertilized eggs, whereas endosymbiont-carrying females remain unaffected and fully compatible. CI benefits the endosymbionts by decreasing the reproductive success of females that do not carry the endosymbionts, facilitating endosymbiont spread within host populations (Turelli & Hoffmann, 1991). However, this benefit bestowed upon endosymbiont-carrying hosts imposes a reproductive cost on endosymbiont-free females, leading to a conflict between host and endosymbiont (Wedell, 2020; Werren, 2011). Moreover, the occupation of the same host by multiple different maternally transmitted endosymbionts (including CI-inducing endosymbionts) may result in competition between these endosymbionts and complex interactions between them and their hosts.

The reproductive consequences of CI are well-documented (Doremus & Hunter, 2020; Turelli & Hoffmann, 1991; Werren et al., 2008), and its influence on mating behavior and mate choice has received increasing attention across diverse host species (Amini et al., 2024; Arbuthnott et al., 2016; Bagheri et al., 2019; Champion de Crespigny & Wedell, 2007; Koukou et al., 2006; Miller et al., 2010; Vala et al., 2004). Similarly, mating behavioral responses have also been reported for other endosymbiont-facilitated reproductive effects such as feminization (Beltran-Bech & Richard, 2014; Moreau et al., 2001; Richard, 2017) and male killing (Charlat et al., 2007). However, most research has focused on hosts carrying only one endosymbiont rather than multiple endosymbionts. Therefore, while many hosts can carry multiple endosymbionts that independently cause CI (Morrow & Riegler, 2021; Nguyen et al., 2017), the interactive effects of multiple endosymbiont species on host mating behavior and mate choice are completely unknown. Specifically, it remains unclear whether and how conflicts between host and multiple endosymbionts drive chemical and behavioral adaptations that enable host individuals to recognize and avoid incompatible mates, thereby reducing the risk of CI-associated reproductive failure. One potential but largely unexplored mechanism for this is the recognition of endosymbiont-induced changes in cuticular hydrocarbons (CHCs), which are important key chemical signals in arthropod mate recognition and selection (Blomquist & Bagnères, 2010; Blomquist & Ginzel, 2021; Howard & Blomquist, 1982; Otte et al., 2018).

Several studies have investigated the role of microbial symbionts in modulating chemical cues in arthropods (Engl & Kaltenpoth, 2018; Hertaeg et al., 2021), and some of these have focused on *Wolbachia* (Richard, 2017; Ronchetti et al., 2023; Schneider et al., 2019). However, the impact of endosymbionts on sexual selection through CHC alterations remains largely unexplored (Schneider et al., 2019). Moreover, no studies have examined the interactive role of multiple endosymbiont species such as *Wolbachia* and *Cardinium* in shaping host mate choice and chemical signaling, despite evidence that their coexistence within the same host populations can influence host fitness and population dynamics (Hubert et al., 2021, 2025; Li et al., 2020; Ros & Breeuwer, 2009; White et al., 2009).

Kelly’s citrus thrips (*Pezothrips kellyanus*), a significant pest of citrus globally, native to Australia and invasive in other parts of the world (Nguyen et al., 2016), provides a valuable model system to study the interplay between CI-inducing endosymbionts and hosts in host sexual selection. This haplodiploid insect species is naturally associated with CI-inducing *Cardinium* and *Wolbachia* (Nguyen et al., 2017), with almost all individuals of all populations carrying *Cardinium*, while *Wolbachia* prevalence varies widely (0%–100%) across populations (Katlav et al., 2024; Nguyen et al., 2016). Notably, while the two endosymbionts independently cause CI in *P. kellyanus* (Nguyen et al., 2017), they differ in their effects on fitness traits such as sex allocation, resource investment, and reproductive outcomes, with *Cardinium* generally providing benefits, whereas *Wolbachia* is moderately costly to the host (Katlav et al., 2022a; Nguyen et al., 2017). These contrasting effects may explain why *Wolbachia* is less prevalent in *P. kellyanus* populations compared to *Cardinium* (Katlav et al., 2024), despite *Wolbachia*'s stronger CI effect in this host (Nguyen et al., 2017). Other factors may also contribute to this difference: *Wolbachia* has more recently been acquired by this host species and may currently be spreading (Katlav et al., 2024). Furthermore, a recent study has shown that *Cardinium*-carrying females actively reject males carrying both *Cardinium* and *Wolbachia*, indicative of a behavior that may enable females to avoid CI (Tourani et al., 2024). This previous behavioral study assessed the receptivity of individual females with particular endosymbiont associations (female endosymbiont association types) to particular male endosymbiont association types, rather than examining mate choice where females can choose between different male endosymbiont association types. Furthermore, the mechanisms underlying the recognition of incompatible males remained unclear.

To disentangle the effects of the two phylogenetically diverged endosymbionts *Cardinium* and *Wolbachia* on mate preference and chemical communication in this host species, we used genetically related laboratory lines of *P. kellyanus* with different endosymbiont associations that were established through antibiotic treatment of one laboratory population carrying both *Cardinium* and *Wolbachia* (CW). While *Wolbachia* had previously been readily eliminated to create endosymbiont-free (U) and *Cardinium*-only (C) lines, all attempts to remove *Cardinium* from CW individuals to create a *Wolbachia*-only line were unsuccessful, which confirmed the findings that *Cardinium* provides fitness benefits whereas *Wolbachia* is costly (Katlav et al., 2022a; Nguyen et al., 2017). Using individuals of the CW, C, and U lines, we conducted mating preference experiments to test the interactive effects of female and male endosymbiont associations on mate choice and whether females recognize and discriminate against incompatible males to avoid the cost of CI. We then examined whether the female mate choice, influenced by endosymbiont association, is linked with different CHC profiles of males, and uncovered a mechanism for endosymbiont-mediated mate recognition. By investigating whether endosymbionts alter insect chemical ecology and mating behavior, our study sheds light on the broader evolutionary consequences of endosymbiont-driven sexual selection. It also highlights the evolution of tripartite *Cardinium–Wolbachia*–host interactions, where both host–microbe and microbe–microbe conflicts shape host reproductive strategies. Our findings have implications for pest and vector control strategies that use CI-inducing endosymbionts, such as the incompatible insect technique for suppressing pest populations (Zabalou et al., 2004) or the population replacement strategy for limiting the transmission of arthropod-borne viruses (Hoffmann et al., 2011).

## Methods

### Laboratory thrips lines

The laboratory lines of *P. kellyanus* were maintained under controlled conditions on organic oranges with *Typha* sp. pollen added every second day (Katlav et al., 2021a, 2023). We used three genetically related lines: a line with both *Cardinium* and *Wolbachia* (CW), a line with *Cardinium* only (C), and an endosymbiont-free line (U); the latter two lines derived from individuals of the CW line after antibiotic treatment (Katlav et al., 2022a). To minimize host genetic effects, CW and C females were introgressed with U males for four generations, followed by three generations of maintenance before the experiments commenced. Experimental cohorts of age-controlled virgins were obtained using synchronized oviposition, standardized rearing conditions, and separation of sex at the pupal stage (Katlav et al., 2021a). Full details about line maintenance, endosymbiont diagnostics, and experimental cohort establishment are provided in Supplementary Methods 1 (including the list of PCR primers in Table S1).

### Mate choice experiments involving live females and males

For mating preference tests, we used Petri dishes (30 mm diameter, 15 mm height) as mating arenas. For a first set of mate choice experiments, individual virgin females (1–2 days old) of the CW, C, and U lines (30 replicates per line) were placed into the Petri dishes, followed by simultaneous addition of two virgin males with different endosymbiont associations, positioned equidistant from the female. Similarly, in another set of mate choice experiments, individual virgin males of the CW, C, and U lines (20 replicates per line) were placed into the Petri dishes, followed by simultaneous addition of two virgin females with different endosymbiont associations, positioned equidistant from the male. We recorded every stage of each mate choice trial by visually tracking individuals and their interactions. Each trial was also video-recorded and reviewed to confirm the accuracy of observations eliminating the need for physical marking of individuals, which could have introduced confounding effects.

For each replicate, we used clean fine paint brushes for the transfer of individuals and new Petri dishes to prevent any potential CHC contamination. Brushes were cleaned by immersion in bleach, followed by washing with dishwashing liquid, 70% ethanol and distilled water, each step for approximately 1 min. To eliminate position effects, we alternated the relative position of individuals in the Petri dishes in both female and male mating preference experiments. The experiments were conducted within 2 days whereby replicates of all mate choice treatments were equally distributed across each of the 2 days (from 6 a.m. to 6 p.m.). The experiments were conducted in a laboratory with windows and indirect sunlight, as well as additional standard laboratory lights. Furthermore, we used CW, C, and U males with similar-sized forewings and applied the same size selection for females. To confirm this, forewing length of all individuals was measured under a stereomicroscope using a calibrated ocular micrometer. Since forewing size is a reliable proxy for overall body size (Nakao, 1994, Katlav et al., 2021a), this size-based selection controlled for any previously reported size differences across endosymbiont associations (Katlav et al., 2022a), ensuring that our observations were independent of size effects.

It has previously been shown that *P. kellyanus* males approach females for copulation, and females either accept or reject them after a behavioral sequence of physical interactions (Tourani et al., 2024). However, while males generally initiate courtship by approaching females, females occasionally also approach males. Therefore, we defined the “first mating approach” as the first physical contact irrespective of which sex initiated it, and recorded for each female the first male (with a certain endosymbiont association) to physically contact the female as the first mating approach. If the female accepted the male for mating, we considered this as the female’s preference or mate choice. If the female, however, rejected this male and mated with the other male (with the other endosymbiont association), we scored this as a mate switch and the second male as the female’s ultimate mate choice. Females of *P. kellyanus* generally mate once when provided with two males, although remating can occur under persistent male harassment (Tourani et al., 2024). To standardize outcomes, each mating trial (between either one female and two males or one male and two females) was terminated immediately after the first successful copulation after which individuals were collected for forewing length measurement. We recorded the same parameters in the mate choice experiments involving individual males.

### Mate choice experiments involving individual males and two dead females

Since female acceptance or rejection influences male mate choice outcomes in *P. kellyanus* (Tourani et al., 2024) and other thrips species (Akinyemi et al., 2021), a first contact leading to copulation indicates the male’s preference and its acceptance by the female. However, if the male did not mate with the first female but instead mated with the other female, it remains unclear whether this resulted from the first female’s rejection or the male’s preference. Therefore, we conducted an experiment using freeze-killed females, a behaviorally relevant approach given documented necrophilic behavior in thrips species (Akinyemi et al., 2021). For this, virgin CW, C, and U females were killed by freezing at − 20°C for 15 min and then kept at room temperature for 15 min prior to the mating experiment. Individual CW, C, and U males (*n* = 40 per line) were presented with a choice between two dead females with different endosymbiont associations in a 30-mm Petri dish. Female positions were alternated to avoid position effects. For each mating trial, we recorded the first mating approach (defined as the first physical contact with either dead female), any mate switch (a change from the first-contacted dead female to the other dead female), and the ultimate mate choice (when the dead female was mounted as part of a copulation attempt).

### Chemical analyses of CHCs

To examine the role of CHCs in mate recognition, we extracted and analyzed CHC profiles of CW, C, and U males. All males used for CHC extraction were virgin, age-matched adults (1–2 days old) reared under identical conditions. For each male type 31–33 samples were analyzed, each consisting of 200–300 pooled males. CHCs were extracted in *n*-hexane and analyzed using gas chromatography–mass spectrometry (GC–MS). The identification of the chemical compounds was based on diagnostic fragmentation patterns and retention indices relative to *n*-alkane standards (Carlson & Brenner, 1988). Full extraction protocols, thermal settings, and spectral processing details are provided in the Supplementary Methods 2.

### Statistical analyses of the mate choice experiments

A paired-samples t-test was conducted to compare the forewing size of the males used across the 2 days of the first set of mate choice experiments, as well as the forewing size of the females in the 2 days of the second set of mate choice experiments. No statistically significant differences in forewing size were found among endosymbiont association types for males or females used in the mate choice experiments (Table S2). A chi-square test of independence (Pearson chi-square test) in SPSS v29 was used to evaluate whether there was a significant difference in mate choice outcomes between the 2 days across all experiments, and no statistically significant differences were found (Table S3) and, hence, the 2 days were combined for the statistical analyses. Chi-square tests of independence were further used to determine whether male endosymbiont association influenced female mate choice outcomes or female endosymbiont association influenced male mate choice outcomes.

### Statistical analyses of CHCs

The peak area of each CHC was normalized against the peak area of the internal standard (dodecane) and log_10_ transformed. Then we used one-way analysis of variance (ANOVA) to compare the standardized peak areas of individual CHC compounds among CW, C, and U males. Mean values (± standard error) were calculated for each compound within each male type. Statistically significant differences among male endosymbiont association types were determined using Tukey’s HSD post-hoc test. Discriminant function analysis (DFA) (Weddle et al., 2013) was used to assess whether the CHC profiles of males with different endosymbiont associations can be distinguished. For this, tridecane was excluded from the analysis as it was consistently absent in two of three endosymbiont association types. Wilks’ Lambda tests were used to evaluate the overall significance of group separation, and cross-validation was used to evaluate the accuracy and predictive ability of the DFA. To identify which CHC compounds contributed most to the discrimination between endosymbiont association types, we examined the structure matrix and considered compounds with absolute loadings of |0.20| as meaningful contributors.

## Results

### Mate choice between an individual female and two males with different endosymbiont associations

Across endosymbiont associations (CW, C, and U), we tested the mate choice outcomes of individual virgin females when provided with a choice between two virgin males carrying different endosymbiont associations (Table 1). The males approached the females (first mating approach), who then either accepted the first male with a particular endosymbiont association for mating or rejected this male to switch to the other male with another endosymbiont association (mate switch), which led to a successful mating (ultimate mate choice).

**Table 1.** Mate choice outcomes between individual females and two males with different endosymbiont associations (U endosymbiont-free; C *Cardinium*; CW *Cardinium* and *Wolbachia*).

		First mating approach					Mate switch					Ultimate mate choice				
Female (*n* = 30)	Male A (*n* = 30) and B (*n* = 30)	Contact (*n*)	%	Pearson chi-square	df	*p*	Change decision (*n*)	%	Pearson chi-square	df	*p*	Choice (*n*)	%	Pearson chi-square	df	*p*
U	U	15	50	0.000	1	1.000	4	26.7	0.186	1	0.666	14	46.7	0.133	1	0.715
	C	15	50				3	20				16	53.3			
U	C	11	36.7	2.133	1	0.144	4	36.4	0.835	1	0.361	11	36.7	2.133	1	0.144
	CW	19	63.3				4	21.1				19	63.3			
U	U	13	43.3	0.533	1	0.465	6	46.2	1.697	1	0.193	11	36.7	2.133	1	0.144
	CW	17	56.7				4	23.5				19	63.3			
C	U	12	40	1.200	1	0.273	7	58.3	7.646	1	**0.006**	7	23.3	8.533	1	**0.003**
	C	18	60				2	11.1				23	76.7			
C	C	13	43.3	0.533	1	0.465	0	0	17.543	1	**<0.001**	26	86.7	16.133	1	**0.001**
	CW	17	56.7				13	76.5				4	13.3			
C	U	13	43.3	0.533	1	0.465	3	23.1	6.652	1	**0.01**	22	73.3	6.533	1	**0.011**
	CW	17	56.7				12	70.6				8	26.7			
CW	U	17	56.7	0.533	1	0.465	2	11.8	1.639	1	0.201	15	50	0.000	1	1.000
	C	13	43.3				0	0				15	50			
CW	C	12	40	1.200	1	0.273	0	0	0.690	1	0.406	14	46.7	0.133	1	0.715
	CW	18	60				1	5.6				16	53.3			
CW	U	11	36.7	2.133	1	0.144	0	0	-	-	-	11	36.7	2.133	1	0.144
	CW	19	63.3				0	0				19	63.3			

The percentages of the first mating approach and the ultimate mate choice were calculated across all males (A and B) provided to individual females, whereas the percentages of mate switch were calculated for A and B males separately. Significant differences are displayed in bold.

#### First mating approach

Across all nine mate choice treatments, females were randomly approached by either male type (Table 1).

#### Mate switch

When C females were given a choice between a C and a CW male, 76.5% of those initially approached by a CW male switched to mate with a C male, whereas none of the females first approached by a C male switched to a CW male (χ² = 17.543, df = 1, *p* < 0.001). When C females were provided with both a C and a U male, 58.3% of females first approached by a U male switched to mate with a C male, while only 11.1% of those first approached by a C male switched to a U male (χ² = 7.646, df = 1, *p* = 0.006). Finally, when C females were presented with a U and a CW male, 70.6% of those initially approached by a CW male switched to mate with a U male, whereas only 23.1% switched from a U to a CW male (χ² = 6.652, df = 1, *p* = 0.010). In contrast, no significant mate switches were observed in U or CW females provided with the same mating options as C females (Table 1).

#### Ultimate mate choice

When C females were provided with both a C and a CW male, 86.7% of females eventually chose to mate with C over CW males (χ² = 16.133, df = 1, *p* < 0.001). When C females were provided with both a C and a U male, 76.7% of females eventually chose to mate with C males over U males (χ² = 8.533, df = 1, *p* = 0.003). When C females were provided with both a U and a CW male, 73.3% of females eventually chose to mate with U over CW males (χ² = 6.533, df = 1, *p* = 0.011). However, no significant preference in mate choice was observed in U or CW females provided with the same mating options as C females (Table 1).

**Figure 1.**
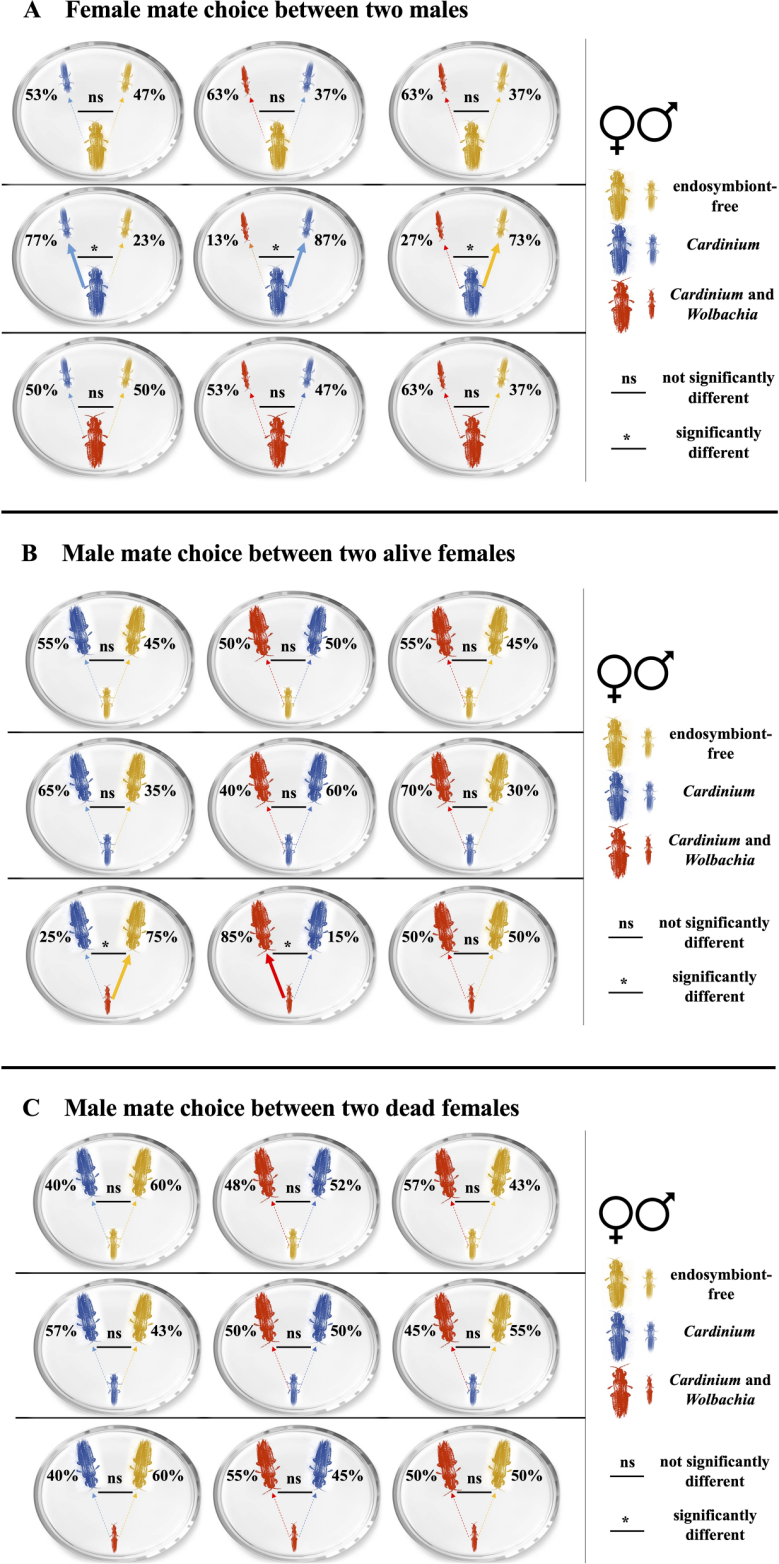
Mate choice of endosymbiont-free (U), *Cardinium* (C), and *Cardinium* and *Wolbachia* (CW) individuals of *Pezothrips kellyanus*. (A) Ultimate mate choice outcome after providing individual virgin females with a choice between two virgin males with different endosymbiont associations. (B) Ultimate mate choice outcome after providing individual virgin males with a choice between two live virgin females with different endosymbiont associations. (C) Ultimate mate choice outcome after providing individual virgin males with a choice between two dead virgin females with different endosymbiont associations. Arrows point from the chooser to the chosen, with solid arrows indicating statistically significant preferences (* signifies *p* < 0.05; ns indicates no significant difference). Percentages represent the proportion of choices directed toward each endosymbiont association type.

In summary, only in mate choice treatments involving a C female, mate switches, and ultimate mate choices were significantly affected by the males’ endosymbiont association. In contrast, in all mate choice treatments involving U or CW females, no significant mate switches occurred, and their ultimate mate choices were irrespective of the males’ endosymbiont associations (Figure 1A).

### Mate choice between an individual male and two live females with different endosymbiont associations

We then tested across endosymbiont associations the mate choice outcomes of individual virgin males when provided with a choice between two live virgin females with different endosymbiont associations (Table 2).

**Table 2.** Mate choice outcomes between individual males and two live females with different endosymbiont associations (U endosymbiont-free; C *Cardinium*; CW *Cardinium* and *Wolbachia*).

		First mating approach					Mate switch					Ultimate mate choice				
Male (*n* = 20)	Female A (*n* = 20) and B (*n* = 20)	Contact (*n*)	%	Pearson chi-square	*df*	*p*	Change decision (*n*)	%	Pearson chi-square	*df*	*p*	Choice (*n*)	%	Pearson chi-square	*df*	*p*
U	U	8	40	0.800	1	0.371	0	0	0.702	1	0.402	9	45	0.200	1	0.655
	C	12	60				1	8.3				11	55			
U	C	12	60	0.800	1	0.371	2	16.7	1.481	1	0.224	10	50	0.000	1	1.000
	CW	8	40				0	0				10	50			
U	U	9	45	0.200	1	0.655	0	0	-	-	-	9	45	0.200	1	0.655
	CW	11	55				0	0				11	55			
C	U	7	35	1.800	1	0.180	0	0	-	-	-	7	35	1.800	1	0.180
	C	13	65				0	0				13	65			
C	C	12	60	0.800	1	0.371	0	0	-	-	-	12	60	0.800	1	0.371
	CW	8	40				0	0				8	40			
C	U	6	30	3.200	1	0.074	0	0	-	-	-	6	30	3.200	1	0.074
	CW	14	70				0	0				14	70			
CW	U	13	65	1.800	1	0.180	0	0	4.127	1	**0.042**	15	75	5.000	1	**0.025**
	C	7	35				2	28.6				5	25			
CW	C	4	20	7.200	1	**0.007**	1	25	4.211	1	**0.04**	3	15	9.800	1	**0.002**
	CW	16	80				0	0				17	85			
CW	U	10	50	0.000	1	1.000	0	0	-	-	-	10	50	0.000	1	1.000
	CW	10	50				0	0				10	50			

The percentages of the first mating approach and the ultimate mate choice were calculated across all females (A and B) provided to individual males, whereas the percentages of mate switch were calculated for A and B females separately. Significant differences are displayed in bold.

#### First mating approach

In all nine mate choice treatments, the male’s initial approach to either female type was random (Table 2), except in the mate choice treatment involving CW males with a C and a CW female, where CW males showed a significant preference, as 80% first approached the CW female (χ² = 7.200, df = 1, *p* = 0.007).

#### Mate switch

When CW males were provided with both a U and a C female, 28.6% of those that had initially approached a C female were rejected and switched to mate with a U female, while none of the males that had first approached a U female were rejected (χ² = 4.127, df = 1, *p* = 0.042). When CW males were given a choice between a C and a CW female, 25% of those that initially approached a C female were rejected and subsequently switched to a CW female, whereas none were rejected by a CW female (χ² = 4.211, df = 1, *p* = 0.040). No significant mate switches were observed in U or C males when presented with the same mating options as CW males (Table 2).

#### Ultimate mate choice

In the mate choice treatment involving individual CW males provided with both a C and a CW female, a significant effect was observed in that 85% of CW males were ultimately accepted for mating by a CW female (χ² = 9.800, df = 1, *p* = 0.002). Moreover, in the mate choice treatment involving individual CW males provided with both a U and a C female, 75% of CW males were ultimately accepted by a U female (χ² = 5.000, df = 1, *p* = 0.025). In contrast, in mate choice treatments involving U and C males, no significant preference in the ultimate mate choice was observed (Table 2).

In summary, only in mate choice treatments involving a CW male, mate switches, and ultimate mate choices were significantly affected by the females’ endosymbiont associations. In contrast, in all other mate choice treatments involving U and C males, no significant mate switches occurred, and ultimate mate choices were irrespective of the females’ endosymbiont associations (Figure 1B).

### Mate choice between an individual male and two dead females with different endosymbiont associations

When interpreting male mate choice outcomes, it is crucial to consider that intrinsic male mate choice can be masked by the females’ mate choice, such as the lower mating receptivity of C females toward incompatible CW males when compared to other female types. Therefore, true male preference cannot be fully elucidated in experiments with live females that exhibit their own mating preferences. To overcome this, we harnessed the necrophilic behavior of *P. kellyanus* to perform an experiment in which CW, C, and U males were individually given a choice between two freeze-killed females representing any two of the three endosymbiont association types. We then found that across all nine mate choice treatments with dead females, the males’ initial approach did not differ between the two different dead female types (Table 3). Similarly, neither mate switching nor ultimate mate choice was influenced by the endosymbionts in the dead females provided (Figure 1C).

**Table 3.** Mate choice outcome between individual males and two dead females with different endosymbiont associations (U endosymbiont-free; C *Cardinium*; CW *Cardinium* and *Wolbachia*).

		First mating approach					Mate switch					Ultimate mate choice				
Male (*n* = 40)	Female A (*n* = 40) and B (*n* = 40)	Contact (*n*)	%	Pearson chi-square	*df*	*p*	Change decision (*n*)	%	Pearson chi-square	*df*	*p*	Choice (*n*)	%	Pearson chi-square	*df*	*p*
U	dead U	24	60	1.6	1	0.206	2	8.3	0.0	1	1.000	24	60	1.6	1	0.206
	dead C	16	40				2	12.5				16	40			
U	dead C	23	57.5	0.9	1	0.343	4	17.4	0.667	1	0.414	21	52.5	0.1	1	0.752
	dead CW	17	42.5				2	11.8				19	47.5			
U	dead U	19	47.5	0.1	1	0.752	3	15.8	1	1	0.317	17	42.5	0.9	1	0.343
	dead CW	21	52.5				1	4.8				23	57.5			
C	dead U	17	42.5	0.9	1	0.343	2	11.8	0.000	1	1.0	17	42.5	0.9	1	0.343
	dead C	23	57.5				2	8.7				23	57.5			
C	dead C	21	52.5	0.1	1	0.752	4	19.1	0.667	1	0.414	20	50	0.000	1	1.000
	dead CW	19	47.5				2	10.5				20	50			
C	dead U	20	50	0.000	1	1.000	3	15	1	1	0.317	22	55	0.4	1	0.527
	dead CW	20	50				1	5				18	45			
CW	dead C	19	47.5	0.1	1	0.725	6	31.6	0.302	1	0.583	18	45	0.400	1	0.527
	dead CW	21	52.5				5	23.8				22	55			
CW	dead U	22	55	0.4	1	0.527	3	13.6	1.237	1	0.266	24	60	1.6	1	0.206
	dead C	18	45				5	27.8				16	40			
CW	dead U	19	47.5	0.1	1	0.725	2	10.5	0.2	1	0.655	20	50	0.000	1	1.000
	dead CW	21	52.5				3	14.3				20	50			

The percentages of the first mating approach and the ultimate mate choice were calculated across all dead females (A and B) provided to individual males, whereas the percentages of mate switch were calculated for A and B females separately.

### Chemical analyses of CHCs of males

Significant differences were found in the standardized peak area of multiple CHC compounds across males of the three endosymbiont association types (Table S4). Tukey’s post-hoc tests revealed a complex pattern, with some CHC compounds being significantly higher and some significantly lower across the three endosymbiont association types. Notably, tridecane was significantly more abundant in CW males compared to C and endosymbiont-free males, which both completely lacked tridecane (*p* < 0.0001). Similarly, ethyl-cyclooctadecane was significantly different across all three endosymbiont association types (*p* < 0.0001), with CW males having the lowest relative abundance of this compound.

### Discriminant function analysis of CHC profiles of males

Given the complex differences detected for CHC compounds in males of the three endosymbiont association types, we further analyzed their CHC profiles using a multivariate approach. DFA identified two significant discriminant functions (DF) (Table 4), explaining 100% of the variance in the dataset (Eigenvalue for DF1 40.248, 88.75% variance explained; Eigenvalue for DF2 5.102, 11.25% variance explained). The canonical correlation for DF1 was 0.988, while DF2 had a canonical correlation of 0.914, indicating strong discriminatory power. A multivariate test of significance confirmed that the DF1 and DF2 were significantly different across the different male endosymbiont association types (Wilks’ Lambda for DF1 = 0.004, Chi-square = 461.612, df = 40, *p* < 0.001; Wilks’ Lambda for DF2 = 0.164, Chi-square = 151.025, df = 19, *p* < 0.001).

**Table 4.** Discriminant function analysis (DFA) examining whether cuticular hydrocarbon (CHC) profiles differ significantly across endosymbiont association types.

CHC compounds	DF1	DF2
Ethyl-cyclooctadecane,	**0.508**	**0.256**
(1-decylundecyl)-cyclohexane	**0.331**	**0.204**
Heneicosyl-cyclopentane	−0.027	0.104
1-nonacosene	0.051	−0.026
Docosyl-cyclohexane	−0.124	−0.147
Cyclotetradecane	0.070	−0.038
11-decyl-docosane	0.035	0.176
Z-12-pentacosene	−0.053	0.163
6,9-dimethyl-tetradecane	**0.347**	−0.167
Octadecyl-cyclohexane	−0.050	**0.285**
2,6,7-trimethyl-decane	−0.015	0.059
(E)-5-eicosene	−0.055	0.085
11-tricosene	0.032	0.128
2,6,10,14-tetramethyl-pentadecane	0.142	0.085
2,6-dimethyl-octadecane	0.065	0.059
13-undecyl-pentacosane	−0.029	0.096
1,3-bis(1,1-dimethylethyl)-benzene	**0.266**	0.090
1-tetradecene	0.028	0.097
2-methyl-nonacosane	−0.031	0.090
2-methyl-dodecane	−0.031	0.094

The DFA has been conducted without tridecane, which was absent from two of the three endosymbiont association types. Discriminatory function (DF1 and DF2) loadings of |0.20| or greater are considered biologically significant (in bold).

**Figure 2.**
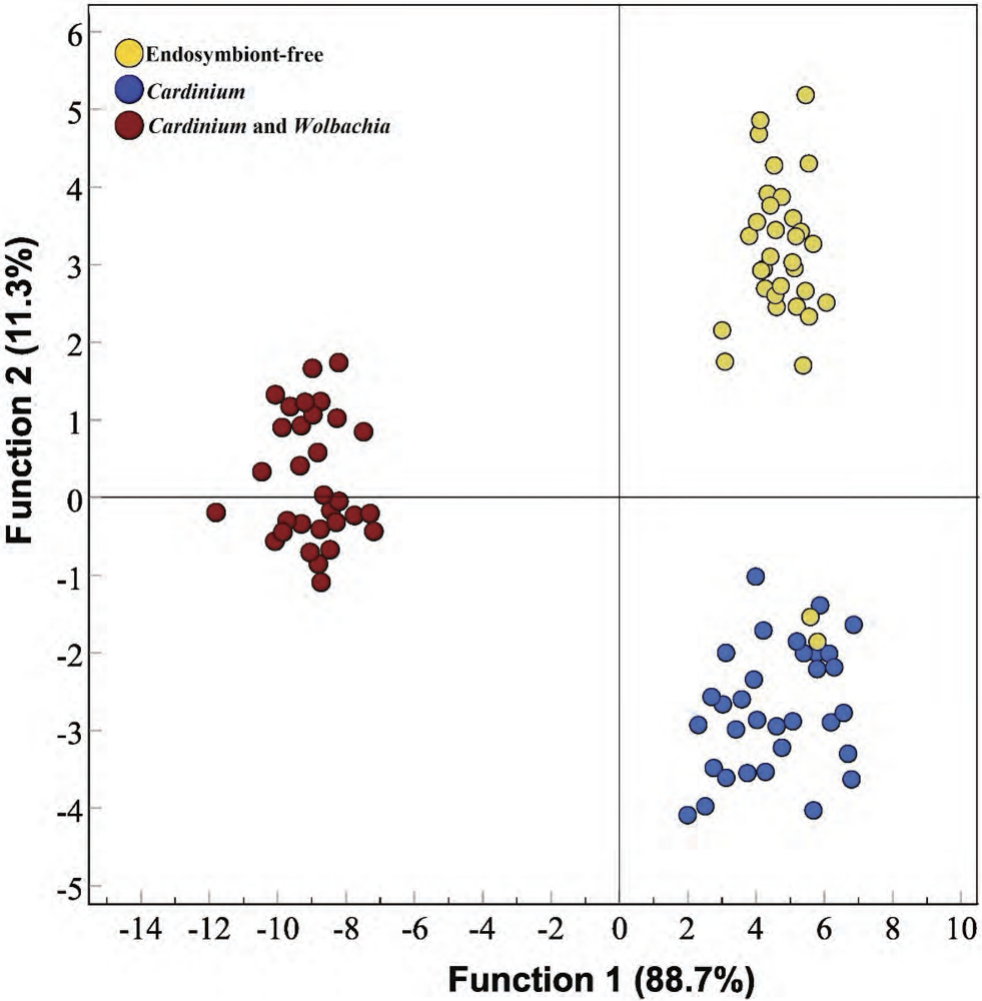
Discriminant function analysis (DFA) scatter plot showing the classification of endosymbiont-free (U), *Cardinium* (C), and *Cardinium* and *Wolbachia* (CW) males based on cuticular hydrocarbon (CHC) profiles.

The four CHC compounds ethyl-cyclooctadecane, (1-decylundecyl)-cyclohexane, 6,9-dimethyl-tetradecane and 1,3-bis(1,1-dimethylethyl)-benzene weighted positively to DF1, whereas the three CHC compounds ethyl-cyclooctadecane, (1-decylundecyl)-cyclohexane and octadecyl-cyclohexane weighted positively to DF2 (Table 4). Inspection of the DFA plot showed that DF1 primarily separated CW males from the other endosymbiont association types: CW males had lower levels of ethyl-cyclooctadecane, (1-decylundecyl)-cyclohexane, 6,9-dimethyl-tetradecane and 1,3-bis(1,1-dimethylethyl)-benzene than U and C males. DF2 predominantly separated C from U males, with U males having higher levels of ethyl-cyclooctadecane, (1-decylundecyl)-cyclohexane and octadecyl-cyclohexane than C males (Figure 2). Our DFA model demonstrated high predictive accuracy, with 96.9% of cross-validated cases correctly classified, and only two of 33 CHC samples of U males were incorrectly classified as C males.

## Discussion

Our study demonstrates that two common and phylogenetically diverged endosymbionts (*Cardinium* and *Wolbachia*) influence mating behavior and sexual selection in a host species in interactive ways. In particular, *P. kellyanus* females with *Cardinium* (C) preferred mating with compatible C males and actively avoided mating with incompatible males carrying both *Cardinium* and *Wolbachia* (CW), and compatible endosymbiont-free (U) males. Further, C females often switched their mate choice to C males after they had first been approached by a CW or an endosymbiont-free male. This discriminatory behavior was not observed in CW and endosymbiont-free females, which did not display any preferences for or against any particular male type (Table 1).

In comparison to females, when individual males were given a choice between two live females with different endosymbiont associations, only CW males showed a preference for approaching and mating with CW females that subsequently accepted them. However, this mate choice outcome needs to be interpreted in the context of the low receptivity of C females toward CW males (Tourani et al., 2024), which could mask intrinsic male mating preferences. Therefore, we provided individual males with a choice between two freeze-killed females with intact CHCs carrying different endosymbiont associations, leveraging previously reported necrophilic behavior of males in thrips (Akinyemi et al., 2021). We then found that males did not have a preference for any endosymbiont association of dead females. Consequently, mate choice outcomes of males were largely influenced by the behavior of potential female mates when they were alive, and not solely by the female’s CHC cues.

Furthermore, the three male types had very distinct CHC profiles that differed qualitatively and quantitatively from each other, indicating that both *Cardinium* and *Wolbachia* affect CHC profiles. In particular, CW males had a CHC profile that was noticeably different from C and U males. This included the presence of tridecane, which was not detected in the two other male types. It is likely that these CHC profile differences enabled C females to recognize and distinguish males based on their endosymbiont association, ultimately guiding their selective mating decisions against mating with incompatible CW males.

### Symbionts affect host sexual selection

Our findings provide strong evidence that *Cardinium* enables females to avoid mates carrying CI-inducing *Wolbachia*, and this *Wolbachia* association is recognizable in the male’s CHC profile. Specifically, C females preferred C males while actively avoiding CI-inducing CW males. Such assortative mating could help reduce the risk of CI and may hinder a CI-driven *Wolbachia* spread in host populations. If females in field populations behave in this way, this may help explain the lower prevalence of *Wolbachia* compared to *Cardinium* in many *P. kellyanus* field populations reported in previous studies (Katlav et al., 2024; Nguyen et al., 2016). However, the fact that *Cardinium* is the only endosymbiont in many populations may also depend on other factors. Mitochondrial population structure of *P. kellyanus* indicates that *Wolbachia* has more recently arrived in this host species (Katlav et al., 2024) and may currently be spreading by inducing CI (Nguyen et al., 2017). Furthermore, *Wolbachia* is moderately costly to *P. kellyanus*, negatively affecting survival under stress, sex allocation, and resource investment (Katlav et al., 2022a).

The finding of *Cardinium*-dependent mate choice that enables females to avoid *Wolbachia*-induced CI points to different potential underlying mechanisms that vary with regard to whether *Cardinium* enables females to recognize the presence of *Wolbachia* in males. One mechanism could be that C females have acquired a *Cardinium*-dependent cognitive capability to detect CHC profile differences in males and then discriminate against incompatible CW males with a distinct CHC profile. This cognitive capability could be mediated by *Cardinium* if it is present in the female’s nervous system, potentially influencing neural processes that govern perception and behavioral decision-making. A recent study has identified potential mechanisms by which *Wolbachia* effector proteins interact with host proteins in the brain and thereby affect female mating receptivity to male courtship and hybrid mating in *Drosophila melanogaster* (Warecki et al., 2025). Like *Wolbachia* (Pietri et al., 2016; Strunov et al., 2022), *Cardinium* has been found in the nervous system of hosts, such as in *Brevipalpus* mites (Kitajima et al., 2007), raising the possibility that it could affect processes in the brain that regulate perception and behavior.

Alternatively, another mechanism could be that both C and endosymbiont-free females can detect the CHC profile of incompatible CW males, and the ability of C females to resist them may stem from the previously demonstrated fitness benefits of *Cardinium* conferred to C females. These fitness benefits include larger body size and greater longevity under stress than CW and endosymbiont-free females (Katlav et al., 2022a). Such enhanced fitness could increase C females’ capacity to resist and escape mating attempts by incompatible CW males. In contrast, endosymbiont-free females have lower overall fitness (Katlav et al., 2022a); therefore they may be less capable to resist mating attempts by incompatible males (Tourani et al., 2024). Furthermore, previous research focusing on compatible matings of *P. kellyanus* found that endosymbiont-free females are more likely than C and CW females to have constrained, highly male-biased offspring sex ratios when mated (Katlav et al., 2021b, 2022a,b). This may further select for reduced resistance of endosymbiont-free females against incompatible matings because they could thereby bypass the risk of CI by keeping their eggs unfertilized. Furthermore, our finding that C females prefer C males over endosymbiont-free males indicates self-recognition of *Cardinium* individuals, and this may be linked to the previous finding that C males are fitter because they have been found to be generally larger and live longer under stress than endosymbiont-free males (Katlav et al., 2022a). This enhanced fitness may enable C males to be persistent and succeed in mating with C females.

Irrespective of whether *Cardinium* enables C females to recognize CW males or enhances these females’ fitness to resist mating attempts by these incompatible males, our findings provide strong evidence that the ability of C females to resist incompatible CW males is mediated by *Cardinium*. This ability is absent in U females. The finding that CW females (which also harbor *Cardinium*) do not resist incompatible CW males may suggest that *Wolbachia* suppresses the *Cardinium*-enabled resistance, further highlighting inter-symbiont conflicts that affect host mating behavior. This finding also confirms the findings that *Wolbachia* increases female mating receptivity and hybrid mating in *D. melanogaster* (Warecki et al., 2025). Similar *Wolbachia* effects have been found in a previous study on *P. kellyanus* where CW females had overall the lowest time to first contact for approaching males and overall lowest resistance frequency to males, while the remating receptivity of CW females was not tested (Tourani et al., 2024). However, this previous study also found that in comparison to *Wolbachia, Cardinium* reduced female receptivity and remating, in particular when facing incompatible males (Tourani et al., 2024). These findings highlight variable endosymbiont effects across different endosymbionts and host associations, as well as competitive interactions that can arise between the two endosymbionts when co-occurring in a host. As observed in the whitefly *Bemisia tabaci* (Lv et al., 2021) and in *Encarsia* parasitoid wasps (Doremus et al., 2020; Wang et al., 2017), *Cardinium* and *Wolbachia* can occur in the same host tissues, including in the reproductive organs. A spatial overlap and occupation of the same ecological niche may result in potential resource competition between these two endosymbionts, which may further disadvantage hosts with both endosymbionts and select against the presence of multiple endosymbionts within individuals, potentially favoring hosts carrying the most rewarding endosymbiont.

Our findings about host–endosymbiont interactions add an important new dimension to observations across diverse animal taxa, where mate choice is influenced by chemical cues that signal mate health and immunocompetence. Although unlikely to be facilitated by microbes (Milinski, 2022), in humans, body odour can convey immunogenetic compatibility, with women preferring men whose major histocompatibility complex (MHC) alleles differ from their own (Wedekind et al., 1995). Female mice use scent cues linked to the MHC to identify males with stronger immune systems (Ehman & Scott, 2001), while female mice avoid odours of parasitized males (Kavaliers & Colwell, 1995). Even in fish, such as the Nile tilapia, females avoid males in advanced stages of bacterial infection (Rossi et al., 2021). By guiding mate choice according to endosymbiont association type, the effects of *Cardinium* on mating behavior found in our study align with broader patterns of symbiont-mediated mating preference. Such endosymbiont-mediated mating preferences may promote host fitness and ensure stable symbiont–host associations. These findings highlight the intricate interplay among endosymbionts with each other and the host’s reproductive strategies and sexual selection.

### Symbionts alter CHC profiles and influence mating behavior

The critical role of CHCs in sexual communication has been extensively documented in many invertebrates (Blomquist & Bagnères, 2010; Howard & Blomquist, 1982). Our study reveals that both *Cardinium* and *Wolbachia* are associated with distinctly different CHC profiles in *P. kellyanus* males. While *Wolbachia*-associated CHC changes have been documented in Diptera, Hymenoptera, and Isopoda (Richard, 2017; Ronchetti et al., 2023; Schneider et al., 2019), our findings provide the first evidence for endosymbiont effects on CHCs in Thysanoptera and that *Cardinium* affects CHC profiles. The *Wolbachia*-associated CHC differences in CW males may be recognized by females as a signal of incompatible males and thereby affect mate choice outcomes.

A stark difference in CHC profiles was the exclusive presence of tridecane in *Wolbachia*-carrying males whereas tridecane was absent in the other male types. Tridecane is widely recognized for its function in defence across diverse insects. In stink bugs, tridecane acts as a major CHC in deterring predators like birds and lizards (Krall et al., 1999; Zarbin et al., 2000). A similar function has been reported in ground beetles (Eisner et al., 1977) and darkling beetles (Geiselhardt et al., 2007). Tridecane, together with tetradecane, was also reported in the CHC profile of moths, albeit their function in the moths is still unclear (Kwadha et al., 2019). In Hymenoptera, it serves as a pheromone component in Japanese carpenter ants (Xu et al., 2023). Interestingly, in Thysanoptera, tridecane was found to be a major CHC compound in *Liothrips jatrophae*; when exposed to tridecane, individuals showed escape and defence behaviors like rapid sideways movement, lifting of the abdomen and droplet secretion at the tip of the abdomen (González-Orellana et al., 2021).

Compounds such as tridecane in the CHC profiles of *P. kellyanus* may also protect against predation; however, this could incur a reproductive cost, as tridecance in CW individuals may act as a repellent for potential mates. Therefore, symbiont-mediated chemical defences may represent a trade-off: while enhancing survival, they may inadvertently disrupt mate signaling, reducing reproductive success. Thus, the role of both endosymbionts in *P. kellyanus* may reflect a complex balance between mate recognition and defence, further shaped by evolutionary conflict between host and the two endosymbionts.

An important question remains: how can endosymbionts affect CHC profiles and synthesis? A previous study demonstrated presence of *Wolbachia* in oenocytes responsible for pheromone and CHC synthesis and implied a direct or indirect interaction of *Wolbachia* with CHC synthesis (Schneider et al., 2019). It will need to be tested whether *Wolbachia* and *Cardinium* are also found in oenocytes of *P. kellyanus*, and whether they influence CHC synthesis directly, for example by encoding CHC synthesis pathway genes, or indirectly by interacting with the host’s CHC synthesis pathway. Future genomic, transcriptomic, and metabolomic analyses of *P. kellyanus*, its two endosymbionts, as well as its microbiome will provide insights into this.

It also needs to be added that our experiments, while providing clear evidence for mating behavioral effects of endosymbionts linked with CHC profile differences, do not allow us to conclude whether any volatile components are also involved. However, the finding that CW males preferentially first approached CW females over C females (Table 2) may suggest a potential involvement of volatiles in the males’ recognition of CW over C females (no other first mating approach combinations showed a significant preference). To investigate the potential involvement of volatiles further, future research should conduct olfactorial Y-tube and electroantennogram experiments as developed for thrips species (de Kogel et al., 1999; Xiu et al., 2022). It may also be useful to manipulate the CHC and volatile profiles experimentally to further demonstrate their roles in the mating behavior of *P. kellyanus* by following perfuming experiment protocols developed for other insects (Weddle et al., 2013).

## Conclusions

Our study reveals that endosymbionts can play a pivotal role in shaping host reproductive strategies and sexual selection by modulating chemical communication and mate choice. We demonstrate that *Cardinium* enables hosts to avoid the risk of reproductive failure due to *Wolbachia*-induced CI. These findings shed light on the evolution of tripartite endosymbiont–host interactions such as the *Cardinium–Wolbachia*–host interaction in our study species, suggesting that symbiont-induced mate preferences may serve as a mechanism to maintain stable symbiont-host associations and limit the invasion of competing endosymbionts occupying the same ecological niche. Furthermore, our research highlights the broader impact of microbial symbionts on insect chemical communication, linking their presence to shifts in CHC profiles that influence mating outcomes. Future research should explore the molecular mechanisms underlying the endosymbionts’ influence on CHC synthesis and cognition. Additionally, it will be important to assess how observed mating patterns affect endosymbiont dynamics and endosymbiont-based pest control strategies. Finally, the potential of intricate interactions between different endosymbionts and their host needs to be considered in the application and development of endosymbiont-based pest and vector management strategies.

## Supplementary Material

qraf044_Supplemental_File

## Data Availability

All data are contained within the main text, additional supporting information (Supplementary Methods 1–2, Tables S1–S4) and at https://doi.org/10.6084/m9.figshare.28889555.v1.
