# Supplementary material for: Common endosymbionts influence host sexual selection by shaping mating preferences via altered chemical communication
Source: Evol Lett. 2025 Dec 8;10(1):77–90. doi: 10.1093/evlett/qraf044 (PMC12870845; doi:10.1093/evlett/qraf044)
Supplement: qraf044_Supplemental_File [file qraf044_supplemental_file.docx]

**Common endosymbionts influence host sexual selection by shaping mating preferences via altered chemical communication**

Amir H. Tourani^1*^, Alihan Katlav^1^, James M. Cook^1^, John Hunt^2^, Shawan Karan^1^, Markus Riegler^1*^

^1^Hawkesbury Institute for the Environment, Western Sydney University, Locked Bag 1797, Penrith, NSW 2751 Australia; ^2^School of Science, Western Sydney University, Locked Bag 1797, Penrith, NSW 2751 Australia

*** Corresponding authors:**

Amir H. Tourani [a.tourani@westernsydney.edu.au](mailto:a.tourani@westernsydney.edu.au)

Markus Riegler [m.riegler@westernsydney.edu.au](mailto:m.riegler@westernsydney.edu.au)

**This file includes**

Supplementary Methods 1 to 2

Tables S1 to S4

Supplementary References

**Supplementary Methods 1**

**Laboratory thrips lines and experimental cohort establishment**

Laboratory lines of *Pezothrips kellyanus* were maintained on organic oranges, with *Typha* sp. pollen added every second day, at 20 ± 1 °C, 70 ± 2% relative humidity and a 16:8 hours (light:dark) photoperiod (Katlav et al., 2021; Nguyen et al., 2017; Tourani et al., 2024). Three laboratory lines, each with a different endosymbiont association, were used: a *Cardinium*-*Wolbachia* (CW) line, initially established using individuals collected from a citrus orchard in Kulnura (New South Wales) in 2017; a *Cardinium*-only line (C); and an endosymbiont-free line (U). The C and U populations were obtained by antibiotic treatment of CW individuals (Katlav et al., 2024). Despite numerous attempts, the establishment of a *Wolbachia-*only line through antibiotic treatment remained unsuccessful (Katlav et al. 2024; Nguyen et al., 2017). The CW line had undergone significant inbreeding before the establishment of the other two lines. Moreover, to minimise genetic variation between the three laboratory lines, C and CW females were introgressed with U males for four generations, followed by three generations of maintenance before the experiments commenced.

To establish similar-aged and density-controlled thrips cohorts, >100 females from each laboratory line were randomly collected and placed into five small containers (20 females per container) with similar-sized fresh oranges for oviposition for 24 hours. To enhance fecundity, *Typha* sp. pollen was sprinkled on the oranges (Katlav et al., 2021; Nguyen et al., 2017; Tourani et al., 2024). Each container was lined with a filter paper at the bottom. At the pupal stage, female and male pupae were carefully separated (Katlav et al., 2021; Nguyen et al., 2017; Tourani et al., 2024) and transferred into individual Petri dishes (30 mm diameter, 15 mm height) lined with moist filter paper. The Petri dishes with the pupae were kept at the same environmental conditions as the lines above until emerged adults (1-2 days old) were used in the experiments.

**PCR diagnostics of endosymbiont association**

The endosymbiont association of the experimental lines was verified using diagnostic PCR. The endosymbiont association was confirmed on ten randomly selected females and five males from each line, both before and after the experiments.

For this, DNA was extracted from individual thrips using a 5% Chelex 100 (Bio-Rad) solution in 10 mM Tris-HCl (pH 8.0), following the protocol established by Kobayashi et al. (2013). Standard PCR protocols, as detailed in Nguyen et al. (2016), were employed to screen for the presence of *Cardinium* and *Wolbachia* using strain-specific primers. The quality of the extracted DNA of each sample was confirmed by successful amplification of the mitochondrial cytochrome c oxidase subunit I (COI) gene. The primers used are listed in Table S1.

**Supplementary Methods 2**

**Chemical analyses of CHCs**

To investigate chemical cues involved in mate choice, CHCs were extracted from CW, C and U males. Each male type was replicated 31-33 times, with each replicate consisting of 40 mg males (200–300 males). CHCs were extracted by immersing the males in a 2 ml vial containing 200 µL of n-hexane for 15 minutes. The CHC-n-hexane extracts were then transferred to new vials with the addition of 20 µL of an internal standard. The solvents were evaporated using a centrifugal evaporator for 8 minutes, after which 40 µL of n-hexane was added to reconstitute the sample for gas chromatography–mass spectrometry (GC-MS) analysis. The extracts were analysed using an Agilent 7890 gas chromatograph coupled with an Agilent 5975 Mass Selective Detector (GC-MS, Agilent, Waldbronn, Germany). The GC, equipped with a DB-5 fused silica capillary column (60 m × 0.25 mm ID, df = 0.25 µm; J&W Scientific, Folsom, United States), was operated in split mode for 2 minutes using a split/split-less injector (2 µL injection volume at 300 °C). Hydrogen was used as the carrier gas with a constant flow rate of 1.4 mL/min. The thermal program had an initial temperature of 36 °C, increasing at 14 °C per minute to 320 °C, followed by an isothermal hold at 320 °C for 20 minutes. Electron ionization mass spectra (EI-MS) were acquired at an ionization voltage of 70 eV, with a source temperature of 250 °C. Data was acquired in Scan mode (40-600 amu) at a speed of 781 u/s. Chromatograms and mass spectra were processed using Agilent MassHunter Unknowns Analysis (version 12.1; Agilent Technologies, Santa Clara, California). To determine retention indices, 1 alkane standards (C7-C40; Sigma Aldrich, St. Louis, Missouri) were run alongside the CHC samples. CHC compounds identification was based on compound-specific retention indices and diagnostic fragmentation patterns (Carlson & Brenner, 1988) using the NIST 23 Mass Spectral library.

**Table S1.** Primers used for PCR assays.

| **Primer name** | **Primer sequence (5’ - 3’ )** | **Target gene** | **Reference** |
| --- | --- | --- | --- |
| 81F | TGGTCCAATAAGTGATGAAGAAAC | *wsp*  *Wolbachia* | Zhou et al., 1998 |
| 691R | AAAAATTAAACGCTACTCCA |  |  |
| Wsp for | TGGTCCAATAAGTGATGAAGAAACTAGCTA | *wsp*  *Wolbachia* | Jeyaprakash and Hoy, 2000 |
| Wsp rev | AAAAATTAAACGCTACTCCAGCTTCTGCAC |  |  |
| CLOf1 | GGAACCTTACCTGGGCTAGAATGTATT | *16S rDNA*  *Cardinium* | Duron et al., 2008 |
| CLOr1 | CLOr1 GCCACTGTCTTCAAGCTCTACCAAC |  |  |
| LCO1490 | GGTCAACAAATCATAAAGATATTGG | *COI*  insect | Folmer et al., 1994 |
| HCO2198 | TAAACTTCAGGGTGACCAAAAAATCA |  |  |

**Table S2.** A paired-samples t-test was conducted to compare the forewing size of males of *Pezothrips kellyanus* with different endosymbiont associations (U endosymbiont-free; C *Cardinium*; CW *Cardinium* and *Wolbachia*)*.*in group A and B (first set of mate choice experiments), as well as the average (± SE) forewing size of females and males in group A and B (second set of mate choice experiments).

| **Female (n 30)** | **Male A (n 30)** | **Male B (n 30)** | **t statistic** | **p value** |
| --- | --- | --- | --- | --- |
| U | U (71.13±0.48) | C (70.3±0.43) | 1.115 | 0.274 |
| U | C (70.82±0.50) | CW (71.0±0.58) | -0.250 | 0.805 |
| U | U (70.63±0.53) | CW (70.6±0.54) | 0.039 | 0.969 |
| C | U (71.11±0.49) | C (71.0±0.53) | 0.237 | 0.814 |
| C | C (71.2±0.50) | CW (70.7±0.51) | 0.795 | 0.433 |
| C | U (71.13±0.50) | CW (71.0±0.51) | 0.159 | 0.875 |
| CW | U (71.14±0.44) | C (71.2±0.43) | -0.163 | 0.872 |
| CW | C (70.9±0.50) | CW (71.1±0.47) | -0.223 | 0.825 |
| CW | U (70.96±0.58) | CW (70.67±0.44) | 0.443 | 0.661 |
| **Male (n 20)** | **Female A (n 20)** | **Female B (n 20)** | **t statistic** | **p value** |
| U | U (90.55±0.61) | C (90.8±63) | -0.312 | 0.785 |
| U | U (90.55±0.74) | CW (91.0±0.81) | -0.363 | 0.721 |
| U | C (90.5±0.67) | CW (90.45±0.80) | 0.510 | 0.960 |
| C | U (89.4±0.74) | C (91.6±0.69) | 1.187 | 0.250 |
| C | U (90.95±0.84) | CW (90.9±0.87) | 0.047 | 0.963 |
| C | C (89.85±0.69) | CW (90.0±0.81) | -0.181 | 0.858 |
| CW | U (90.35±0.72) | C (90.7±0.68) | -0.370 | 0.716 |
| CW | U (90.50±0.78) | CW (90.1±0.76) | 0.352 | 0.729 |
| CW | C (90.75±0.83) | CW (91.6±0.68) | -0.928 | 0.365 |

**Table S3.** A chi-square test of independence was used to evaluate whether there was a significant difference between the mate choice outcomes between two days (block 1 and 2) of replicates for each of the two sets of mate choice experiments with *Pezothrips kellyanus* with different endosymbiont associations (U endosymbiont-free; C *Cardinium*; CW *Cardinium* and *Wolbachia*).

| **Treatment [female (male A+ male B)]** |  | | ***T* stat** | ***p* value** |
| --- | --- | --- | --- | --- |
| U (U+C) | Block 1 | Block 2 | 0.000 | 1.0 |
| U (C+CW) | Block 1 | Block 2 | 0.231 | 0.631 |
| U (U+CW) | Block 1 | Block 2 | 0.314 | 0.575 |
| C (U+C) | Block 1 | Block 2 | 0.000 | 1.0 |
| C (C+CW) | Block 1 | Block 2 | 0.29 | 0.591 |
| C (U+CW) | Block 1 | Block 2 | 0.001 | 0.978 |
| CW (U+C) | Block 1 | Block 2 | 0.000 | 1.0 |
| CW (C+CW) | Block 1 | Block 2 | 0.000 | 1.0 |
| CW (U+CW) | Block 1 | Block 2 | 0.574 | 0.449 |
| **Treatment [male (female A+ female B)]** |  | | ***T* stat** | ***p* value** |
| U (U+C) | Block 1 | Block 2 | 0.000 | 1.0 |
| U (C+CW) | Block 1 | Block 2 | 0.808 | 0.369 |
| U (U+CW) | Block 1 | Block 2 | 0.000 | 1.0 |
| C (U+C) | Block 1 | Block 2 | 0.879 | 0.348 |
| C (C+CW) | Block 1 | Block 2 | 0.000 | 1.0 |
| C (U+CW) | Block 1 | Block 2 | 0.238 | 0.626 |
| CW (U+C) | Block 1 | Block 2 | 0.067 | 0.795 |
| CW (C+CW) | Block 1 | Block 2 | 0.000 | 1.0 |
| CW (U+CW) | Block 1 | Block 2 | 0.000 | 1.0 |
| **Treatment [male (dead female A+ dead female B)]** |  | | ***T* stat** | ***p* value** |
| U (U+C) | Block 1 | Block 2 | 0.081 | 0.776 |
| U (C+CW) | Block 1 | Block 2 | 0.000 | 1.0 |
| U (U+CW) | Block 1 | Block 2 | 0.000 | 1.0 |
| C (U+C) | Block 1 | Block 2 | 1.435 | 0.231 |
| C (C+CW) | Block 1 | Block 2 | 0.000 | 1.0 |
| C (U+CW) | Block 1 | Block 2 | 0.101 | 0.751 |
| CW (U+C) | Block 1 | Block 2 | 0.683 | 0.409 |
| CW (C+CW) | Block 1 | Block 2 | 0.021 | 0.885 |
| CW (U+CW) | Block 1 | Block 2 | 0.1 | 0.752 |

**Table S4.** One-way ANOVA results for cuticular hydrocarbon (CHC) compounds across the three endosymbiont association types (U endosymbiont-free, C *Cardinium*, and CW *Cardinium* and *Wolbachia*). This table presents the results of a one-way ANOVA comparing standardised peak area of CHC compounds across the three endosymbiont association types. Also included are the compound retention time represented as the time at which each CHC compound was eluted from the chromatographic column, and the compound formula which indicates the molecular structure of each hydrocarbon. Mean values (± standard error) of the standardised peak area are reported for each CHC compound within each endosymbiont association type. Different capital letters indicate statistically significant differences between endosymbiont association types based on Tukey’s HSD post-hoc test (*p* < 0.05). Compounds with low *p*-values (< 0.05) contribute significantly to CHC differentiation across endosymbiont association types.

| **CHC compounds** | **Compound retention time** | **Compound formula** | **Endosymbiont-free (U)** | ***Cardinium* (C)** | ***Cardinium* and *Wolbachia* (CW)** | **F-stat** | **df** | ***p*-value** |
| --- | --- | --- | --- | --- | --- | --- | --- | --- |
| Tridecane | 11.23 | C13H28 | 0.00 ± 0.00 B | 0.00 ± 0.00 B | 1.70 ± 0.01 A | 34448.88 | 2 | <0.0001 |
| Ethyl-cyclooctadecane | 18.23 | C20H40 | 1.96 ± 0.02 A | 1.78 ± 0.04 B | 0.95 ± 0.01 C | 497.76 | 2 | <0.0001 |
| (1-decylundecyl)-cyclohexane | 21.42 | C27H54 | 1.21 ± 0.02 A | 1.07 ± 0.03 B | 0.59 ± 0.01 C | 215.26 | 2 | <0.0001 |
| Heneicosyl-cyclopentane | 21.43 | C26H52 | 1.23 ± 0.01 B | 1.19 ± 0.01 C | 1.24 ± 0.02 A | 3.93 | 2 | 0.0231 |
| 1-nonacosene | 22.59 | C29H58 | 1.06 ± 0.02 A | 1.20 ± 0.31 A | 0.43 ± 0.01 B | 5.04 | 2 | 0.0084 |
| Docosyl-cyclohexane | 22.78 | C28H56 | 0.64 ± 0.02 C | 0.73 ± 0.02 B | 0.88 ± 0.02 A | 33.86 | 2 | <0.0001 |
| Cyclotetradecane | 14.68 | C14H28 | 0.62 ± 0.02 A | 0.68 ± 0.02 A | 0.38 ± 0.09 B | 9.53 | 2 | 0.0002 |
| 11-decyl-docosane | 23.99 | C32H66 | 0.84 ± 0.02 A | 0.64 ± 0.02 B | 0.64 ± 0.06 B | 9.65 | 2 | 0.0002 |
| Z-12-pentacosene | 20.31 | C25H50 | 0.56 ± 0.02 A | 0.44 ± 0.03 B | 0.59 ± 0.02 A | 11.60 | 2 | <0.0001 |
| 6,9-dimethyl-tetradecane | 13.11 | C16H34 | 0.09 ± 0.02 B | 0.20 ± 0.03 A | -0.42 ± 0.01 C | 231.70 | 2 | <0.0001 |
| Octadecyl-cyclohexane | 20.31 | C24H48 | 0.54 ± 0.02 A | 0.36 ± 0.03 B | 0.53 ± 0.02 A | 24.00 | 2 | <0.0001 |
| 2,6,7-trimethyl-decane | 10.13 | C13H28 | 0.32 ± 0.03 A | 0.27 ± 0.02 A | 0.32 ± 0.03 A | 1.25 | 2 | 0.29 |
| (E)-5-eicosene | 19.11 | C20H40 | 0.13 ± 0.02 B | 0.08 ± 0.02 B | 0.18 ± 0.02 A | 7.33 | 2 | 0.0011 |
| 11-tricosene | 19.10 | C23H46 | 0.10 ± 0.02 A | -0.05 ± 0.03 B | -0.07 ± 0.06 B | 5.84 | 2 | 0.0041 |
| 2,6,10,14-tetramethyl-pentadecane | 14.84 | C19H40 | 0.08 ± 0.01 A | -0.02 ± 0.03 A | -0.37 ± 0.06 B | 39.42 | 2 | <0.0001 |
| 2,6-dimethyl-octadecane | 16.49 | C20H42 | -0.06 ± 0.01 A | -0.14 ± 0.03 A | -0.33 ± 0.08 B | 8.66 | 2 | 0.0004 |
| 13-undecyl-pentacosane | 26.10 | C36H74 | -0.02 ± 0.02 A | -0.07 ± 0.01 B | -0.01 ± 0.02 A | 3.77 | 2 | 0.0266 |
| 1,3-bis(1,1-dimethylethyl)-benzene | 10.71 | C14H22 | -0.08 ± 0.02 A | -0.14 ± 0.02 A | -0.52 ± 0.03 B | 134.73 | 2 | <0.0001 |
| 1-tetradecene | 12.17 | C14H28 | -0.08 ± 0.02 A | -0.18 ± 0.02 A | -0.20 ± 0.05 B | 3.73 | 2 | 0.0277 |
| 2-methyl-nonacosane | 23.09 | C30H62 | -0.11 ± 0.02 A | -0.15 ± 0.01 B | -0.09 ± 0.02 A | 3.67 | 2 | 0.0291 |
| 2-methyl-dodecane | 10.92 | C13H28 | -0.11 ± 0.02 A | -0.16 ± 0.02 B | -0.10 ± 0.02 A | 3.93 | 2 | 0.023 |

**Supplementary References**

Carlson, D.A., & Brenner, R.J. (1988). Cuticular hydrocarbons of North American *Blattella* for identification of all life stages. *Ann. Entomol. Soc. Am.* 81, 711–723.

Duron, O., Bouchon, D., Boutin, S., Bellamy, L., Zhou, L., Engelstädter, J., & Hurst, G.D. (2008). The diversity of reproductive parasites among arthropods: *Wolbachia* do not walk alone. *BMC Biol*. 6, 27.

Folmer, O., Hoeh, W.R., Black, M.B., & Vrijenhoek, R.C. (1994). Conserved primers for PCR amplification of mitochondrial DNA from different invertebrate phyla. *Mol. Mar. Biol. Biotechnol*. 3, 294–299.

Jeyaprakash, A., & Hoy, M.A. (2000). Long PCR improves *Wolbachia* DNA amplification: wsp sequences found in 76% of sixty-three arthropod species. *Insect Mol. Biol.* 9, 393–405.

Kobayashi, K., Yoshimura, J., & Hasegawa, E. (2013). Coexistence of sexual individuals and genetically isolated asexual counterparts in a thrips. *Sci. Rep.* 3, 3286.

Katlav, A., Cook, J.M., & Riegler, M. (2021). Egg size-mediated sex allocation and mating-regulated reproductive investment in a haplodiploid thrips species. *Funct. Ecol.* 35, 485–498.

Katlav, A., Cook, J.M., & Riegler, M. (2022). Common endosymbionts affect host fitness and sex allocation via egg size provisioning. *Proc. R. Soc. B* 289, 20212582.

Nguyen, D.T., Spooner-Hart, R.N., & Riegler, M. (2016). Loss of *Wolbachia* but not *Cardinium* in the invasive range of the Australian thrips species, *Pezothrips kellyanus*. *Biol. Invasions* 18, 197–214.

Nguyen, D.T., Morrow, J.L., Spooner-Hart, R.N., & Riegler, M. (2017). Independent cytoplasmic incompatibility induced by *Cardinium* and *Wolbachia* maintains endosymbiont coinfections in haplodiploid thrips populations. *Evolution* 71, 995–1008.

Tourani, A.H., Katlav, A., Cook, J.M., & Riegler, M. (2024). Mating receptivity mediated by endosymbiont interactions in a haplodiploid thrips species. *Proc. R. Soc. B* 291, 20241564.

Zhou, W., Rousset, F., & O'Neill, S. (1998). Phylogeny and PCR-based classification of *Wolbachia* strains using wsp gene sequences. *Proc. R. Soc. Lond. B* 265, 509–515.
